# A Role for the Host DNA Damage Response in Hepatitis B Virus cccDNA Formation—and Beyond?

**DOI:** 10.3390/v9050125

**Published:** 2017-05-22

**Authors:** Sabrina Schreiner, Michael Nassal

**Affiliations:** 1 Institute of Virology, Technische Universität München/Helmholtz Zentrum München, Ingolstädter Landstr. 1, Neuherberg, D-85764 Munich, Germany; sabrina.schreiner@tum.de; 2 Dept. of Internal Medicine II/Molecular Biology, University Hospital Freiburg, Hugstetter Str. 55, D-79106 Freiburg, Germany

**Keywords:** hepatitis B virus, cccDNA, HBV minichromosome, DNA repair, DNA damage response, HBV cure

## Abstract

Chronic hepatitis B virus (HBV) infection puts more than 250 million people at a greatly increased risk to develop end-stage liver disease. Like all hepadnaviruses, HBV replicates via protein-primed reverse transcription of a pregenomic (pg) RNA, yielding an unusually structured, viral polymerase-linked relaxed-circular (RC) DNA as genome in infectious particles. Upon infection, RC-DNA is converted into nuclear covalently closed circular (ccc) DNA. Associating with cellular proteins into an episomal minichromosome, cccDNA acts as template for new viral RNAs, ensuring formation of progeny virions. Hence, cccDNA represents the viral persistence reservoir that is not directly targeted by current anti-HBV therapeutics. Eliminating cccDNA will thus be at the heart of a cure for chronic hepatitis B. The low production of HBV cccDNA in most experimental models and the associated problems in reliable cccDNA quantitation have long hampered a deeper understanding of cccDNA molecular biology. Recent advancements including cccDNA-dependent cell culture systems have begun to identify select host DNA repair enzymes that HBV usurps for RC-DNA to cccDNA conversion. While this list is bound to grow, it may represent just one facet of a broader interaction with the cellular DNA damage response (DDR), a network of pathways that sense and repair aberrant DNA structures and in the process profoundly affect the cell cycle, up to inducing cell death if repair fails. Given the divergent interactions between other viruses and the DDR it will be intriguing to see how HBV copes with this multipronged host system.

## 1. Introduction

Hepatitis B virus (HBV) is the prototypic member of the hepadnaviruses, a family of small enveloped hepatotropic viruses that replicate their tiny (~3 kb) DNA genomes through reverse transcription. HBV causes acute and chronic hepatitis B; chronic HBV infection puts more than 250 million virus carriers at a greatly increased risk to develop terminal liver disease, i.e., liver fibrosis, cirrhosis and hepatocellular carcinoma (HCC) [1]. While an effective prophylactic vaccine is available since decades and universal vaccination programs have been implemented in many countries, the total number of chronic HBV carriers is still on the rise [2]. HCC is now the third leading [2] if not second leading [3] cause of cancer mortality and >90% of all HCC cases can be attributed, about equally [2], to chronic infection with HBV or hepatitis C virus (HCV). As HCV is an RNA virus and RNA has a limited life-span, blocking replication for a finite time is sufficient to eliminate the virus. This is indeed achieved by recently introduced direct acting antivirals, and chronic hepatitis C can now be cured in most patients [4,5].

**Figure 1:**
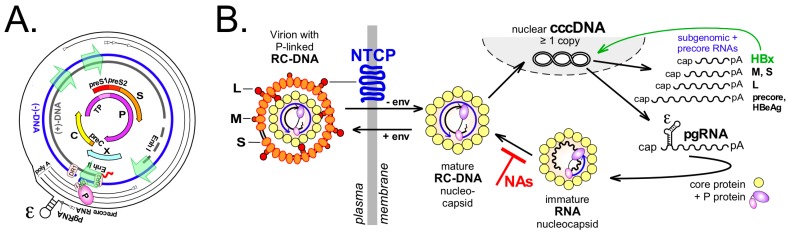
Molecular basics of Hepatitis B Virus (HBV). (**A**) HBV genome organization. Shown from inside to outside are the open reading frames (ORFs) with their designations; the relaxed circular DNA (RC-DNA) strands with the relative positions of direct repeat 1 (DR1), DR2, enhancer I, enhancer II and the four internal promotors (green arrows); and the transcripts with their staggered 5′ ends (arrowheads) and common 3′ polyA ends. ε denotes the RNA stem-loop on pregenomic RNA (pgRNA) that directs co-encapsidation of pgRNA and P protein and protein-primed replication initiation; (**B**) Simplified genome replication cycle. Virus entry is mediated by binding of L protein’s PreS1 domain to Na^+^-taurocholate cotransporting polypeptide (NTCP) [15,16] and additional entry factors (not shown) such as glypican 5 [17]. Nucleocapsids stripped from the envelope transport the P protein-linked RC-DNA to the nucleus where conversion into covalently closed circular DNA (cccDNA) takes place. cccDNA serves as template for the various transcripts, including pgRNA from which core protein and P protein are translated. Via P protein binding to ε, pgRNA is encapsidated (“immature” nucleocapsid) and reverse transcribed into new RC-DNA (“mature” nucleocapsid); this step is inhibited by therapeutic nucleos(t)ide analogs (NAs). Mature progeny nucleocapsids can be enveloped and secreted, or retransport the new RC-DNA to the nucleus to increase cccDNA copy number (“intracellular recycling”). Subgenomic (sg) RNAs act as mRNAs for the envelope proteins and hepatitis B virus X protein HBx which stimulates transcriptional activity of cccDNA (green arrow). Translation of the precore RNA which includes the preC start codon yields precore protein which is processed and secreted as HBeAg.

HBV, by contrast, is a pararetrovirus with an obligatory nuclear phase [6,7,8]. The genome in infectious virions is a protein-linked partially double-stranded (ds) relaxed circular (RC) DNA in which none of the strands is covalently closed (Figure 1A). To serve as a transcription template it is converted into a covalently closed circular (ccc) DNA episome; cccDNA is therefore essential for new viral RNAs, new viral proteins, and virions. In addition, cccDNA provides a long-lived repository for the viral genetic information, thus representing the molecular persistence reservoir of hepadnaviruses. Notably, this function is not coupled to active transcription, making the reservoir, at times, latent and invisible to the immune system [9]. Hence, in many aspects hepadnaviral cccDNA resembles the integrated proviral DNA of retroviruses—except it is not integrated. However, integration can occur, with possibly severe consequences for the host cell [10].

Current treatments for chronic hepatitis B include type I interferons for a fraction of the patients [11], and the better tolerated nucleos(t)ide analogs (NAs) inhibiting reverse transcription for the majority [12]. Either therapy may achieve control of infection but rarely leads to a cure because cccDNA is not directly targeted; even after recovery from acute self-limited hepatitis B cccDNA is not completely eliminated [13,14].

## 2. The Central Role of cccDNA in HBV Replication

The nucleocapsids (core particles) in enveloped HB virions carry relaxed circular DNA (RC-DNA) in which the 5′ end of the minus-strand is covalently linked to the Terminal Protein (TP) domain of the viral P protein (see Figure 1B). Upon infection, the envelope is stripped off; the nucleocapsids released into the host cell’s cytoplasm transport the RC-DNA to the nuclear pore [18], where the capsid structure disintegrates to release the RC-DNA into the nucleus. In a multistep step process, the numerous deviations of RC-DNA from a perfectly double-stranded structure are “repaired”, resulting in cccDNA. Actually as a minichromosome, cccDNA then serves as template for RNA polymerase II-mediated synthesis of the viral transcripts. All are 5′ capped and 3′ polyadenylated; their distinct start sites are defined by four separate promoters (see Figure 1A) but all use the same polyadenylation signal. From the subgenomic transcripts (2.4, 2.1 and 0.8 kb) the envelope proteins L (preS1/preS2/S), M (preS2/S) and S plus hepatitis B virus X protein (HBx) are translated (see Figure 1B). The greater-than-genome-length 3.5 kb transcripts comprise the precore RNA plus the slightly shorter pregenomic RNA (pgRNA). The precore RNA includes the start codon of the preC ORF (see Figure 1A) and thus is translated into the precore protein precursor of the secretory HBeAg. The pgRNA serves as bicistronic mRNA for core protein and P protein and, in addition, as substrate for reverse transcription into new RC-DNA by protein-priming; this establishes the unusual molecular features of RC-DNA [19]. 

Hence, cccDNA is a crucial intermediate in the hepadnaviral replication cycle. Importantly, cccDNA is not directly targeted by current anti-HBV drugs. NAs can potently block reverse transcription of pgRNA and suppress production of new RC-DNA containing virions (see Figure 1B) but not viral transcription and antigen production. As depletion of cccDNA by the interrupted supply of new RC-DNA appears to occur with very slow kinetics, many patients will likely require life-long NA-treatment to keep the virus under control [20,21]. Conversely, very few copies of cccDNA per liver suffice to reactivate full-blown infection once therapeutic and/or immune-mediated control are weakened or lost. Hence, the ultimate goal for a cure of chronic hepatitis B is elimination of the cccDNA reservoir from the patient’s liver—however, many basic issues of cccDNA biology are only beginning to be solved.

## 3. The Actual Transcription Template: Poorly Understood HBV Minichromosome

Nuclear cccDNA is loaded with cellular histone and non-histone proteins forming a nucleosomally organized minichromosome [22,23,24] which apparently also contains viral core protein [23,25] and HBx [26]; HBx positively impacts cccDNA transcriptional activity [27,28,29,30] by de-repressing, perhaps *inter alia*, a restriction by the structural maintenance of chromosomes (Smc) complex Smc5/6 [31,32,33,34].

**Figure 2:**
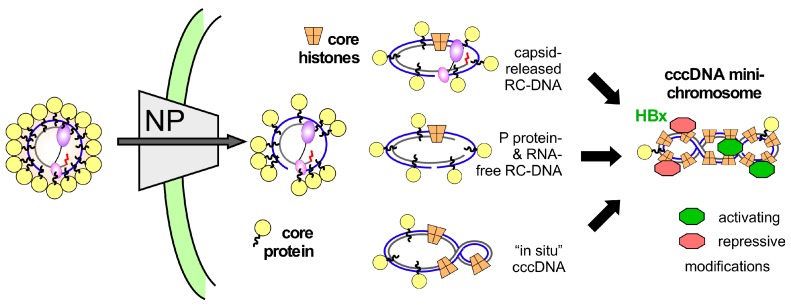
A speculative model for HBV cccDNA minichromosome formation. Interactions at the nuclear pore (NP) cause disintegration of the nucleocapsid structure [18]; however, due to the core protein’s nucleic acid binding C-terminal domain (CTD) (wiggly lines emanating from the yellow spheres symbolizing core protein) not all core protein subunits may be immediately stripped from the RC-DNA. Loading with histones could thus initiate on complexes with still bound P protein and largely unprocessed RC-DNA, or any time later when P protein is released and one or both DNA strands are freshly ligated (termed “in situ” cccDNA in Figure 2). While eventually most molecules will be covalently closed and fully chromatinized, activating and repressive modifications (symbolized by the green and red objects), modulatable by HBx, may be added before this state is reached. In reality, it is likely that on a single cccDNA minichrosome either activating or repressive marks dominate.

However, the pathway from nucleocapsid-borne RC-DNA to chromatinized cccDNA is obscure. While some viruses such as SV40 package their DNA genomes already as histone-associated minichromosomes [35], for hepadnaviruses this must involve the exchange of the DNA-bound core protein against histones, as shown in the conceptual model in Figure 2. Accordingly, the RC-DNA released at the nuclear pore may remain associated with at least some core protein subunits [36], which feature an Arg-rich C terminal domain (CTD) that binds nucleic acids [37,38,39]. Perhaps immediately, or at some time during the multiple steps of RC- to cccDNA conversion, core histones will start being loaded on the DNA and eventually might displace most of the remaining core protein. In addition, the dynamic exchange of histone modifiers, chromatin remodellers and transcription factors will subject the cccDNA to a complex epigenetic regulation of transcriptional activity [40]. As for host chromatin this likely includes DNA methylation, noncoding RNAs and posttranslational histone modifications known as the “histone code” [41,42], albeit with some idiosyncrasies [43]. 

However, the unknown dynamics of core protein replacement by histones on nuclear hepadnaviral DNA raise several issues. A practical caveat concerns chromatin immunoprecipitation (ChIP) assays which may not report exclusively on the cccDNA status, but could include chromatinized non-cccDNA forms. If full displacement of the originally bound core protein by histones is slow, the association of core protein with the minichromosome [23,25] may simply reflect the fortuituous presence of some leftover core protein. Nucleic acid binding by core protein as such is non-sequence specific [44], and promiscuous core protein binding to numerous promoters on chromosomal DNA has been reported [45]. Hence it is unclear how de novo core protein binding would be specific for viral cccDNA. In contrast, during nucleocapsid assembly core protein specificity for the viral nucleic acid is established by the P protein-mediated selective encapsidation of pgRNA and is maintained during RC-DNA formation inside the particle. For core protein subunits surviving uncoating no new specificity mechanism would have to be invoked to explain their association with the minichromosome. Notably, after infection de novo synthesis of core protein is not required for cccDNA transcription [46].

A more fundamental issue implied by the model in Figure 2 is that histone association may not be restricted to cccDNA but might occur as well with RC-DNA (or double-stranded linear DNA (dsL-DNA)). In analogy to recent data showing the rapid loading of histones and subsequently histone marks onto unintegrated retroviral DNA [47] this could even include that chromatinized non-cccDNA molecules are transcribed. Formally, transcripts from non-circularized minus-strand DNA would encode a nearly complete HBx protein lacking just three C terminal amino acids; such a truncation was compatible with functionality of the woodchuck X protein in establishing in vivo infection [48]. At present it is enigmatic how the first HBx molecules are produced when HBx is essential for cccDNA transcription [33]; unconventional mechanisms such as delivery of HBx RNA into the cell [34] yet also formation of HBx transcripts from a non-cccDNA template might not be excluded.

## 4. From P Protein-Linked RC-DNA to cccDNA in Multiple Steps—A Conceptual Overview

Even without the extra complexities of chromatinization, the basic mechanisms of cccDNA formation are not yet well understood, except that each cccDNA molecule arises from a series of biochemical steps that start with an RC-DNA molecule as precursor. The structural differences between the two DNA forms then define the principal modifications RC-DNA must undergo to become cccDNA [19,49]. 

**Figure 3:**
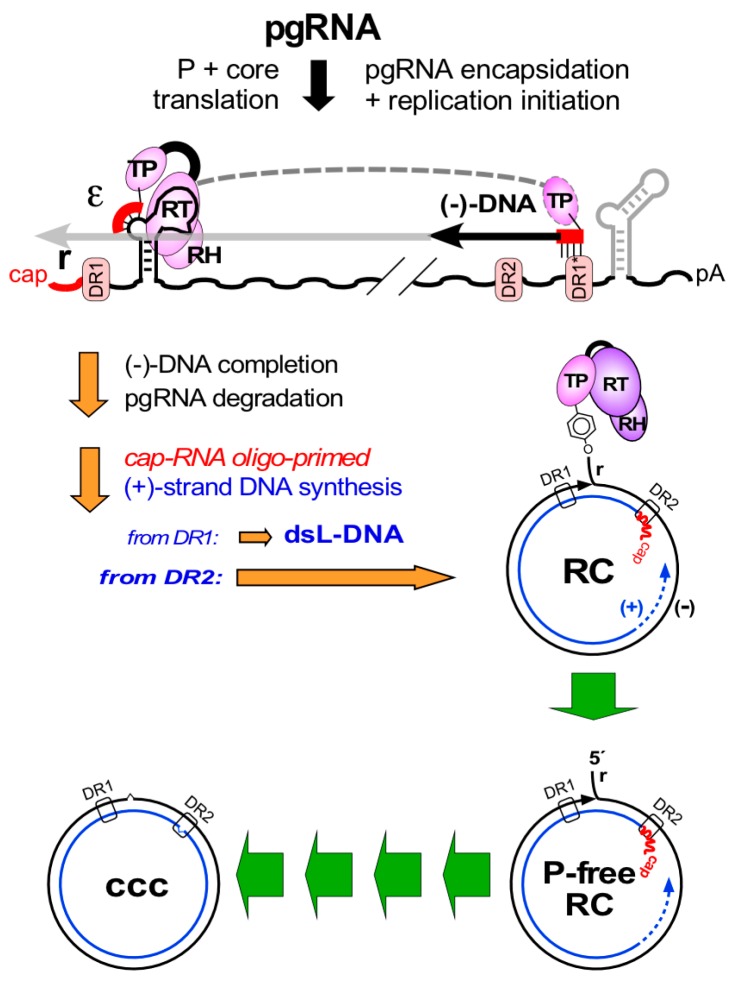
The hepadnaviral genome life-cycle. Protein-primed reverse transcription is initiated by P protein binding to the ε stem-loop on pgRNA, leading to a short ε-templated DNA oligo whose 5′ terminal nt is covalently linked to a Tyr residue in P protein’s terminal protein (TP) domain. Translocation to an acceptor at DR1* allows its extension into slightly overlength minus-strand DNA (carrying the ”r” redundancy), with concomitant pgRNA degradation, except for the capped 5′ terminal end that serves as plus-strand DNA primer. Direct extension from DR1 on yields double stranded linear DNA (dsL-DNA); RC-DNA formation requires primer transfer to DR2 plus an additional template switch (not shown). These steps establish the unusual features of RC-DNA, with non-DNA moieties on both 5′ termini, an overlength minus-strand and an incomplete plus-strand. For cccDNA formation, all peculiarities on RC-DNA must be fixed, both strands must gain exactly unit-length, and the ends must be ligated. The multistep nature of the process is symbolized by the multiple green arrows. One of the predicted intermediates is RC-DNA from which P protein has been released (P-free RC); whether this is the first intermediate as depicted is not known.

Protein-primed reverse transcription of hepadnaviral pgRNA causes RC-DNA to contain several unusual molecular features (see Figure 3). Most obvious is the covalent linkage of P protein to the 5′ terminal nucleotide of (−)-strand DNA. To initiate reverse transcription, P protein binds to a 5′ stem-loop structure on pgRNA, ε, that also acts as RNA encapsidation signal. The phenolic OH group of a Tyr-residue in P protein’s TP domain then mimics the 3’ terminal OH group of a conventional nucleic acid primer and is extended by a few nucleotides, templated by an ε-internal bulge [7,19]. The complex is packaged into newly forming nucleocapsids and the P protein-linked oligonucleotide is translocated to a matching acceptor at the 3′ direct repeat 1* (DR1*). Extension from there yields a slightly overlength minus strand harboring a short ~10 nt terminal redundancy (“r”). Concomitantly, the template RNA is degraded by P protein’s RNase H activity, except for the very 5’ terminal residues harboring DR1. This RNA oligo then serves as nucleic acid primer for plus-strand DNA, either from its original location at 5′ DR1 (“in situ”) yielding dsL-DNA or, as in replication proper, after transfer to DR2, resulting in RC-DNA [7]. Plus-strand synthesis usually does not go to completion in the producer cell, leaving a gap of varying size. Importantly, the linkage of the minus-strand DNA 5′ end to TP remains intact throughout and so does the RNA primer at the 5′ end of plus-strand DNA. Hence, viral particle-associated RC-DNA has 5′ ends consisting of non-DNA moieties, the minus-strand is too long, and the plus-strand is too short (see Figure 3); obviously then, cccDNA formation (green arrow pathway in Figure 3) requires multiple enzymatic activities to fix all these noncanonical features in RC-DNA and eventually ligate the ends.

HBV’s tiny 3 kb genome encodes P protein as the only—albeit multifunctional—enzyme. At least some of the RC-DNA to cccDNA conversion steps could be performed by P protein, foremostly filling-in the gap in plus-strand DNA. However, confirming earlier studies in animal models [50,51,52,53], inhibition of HBV P protein’s DNA polymerase activity did not block cccDNA formation in HepaRG cells [54], HepG2-NTCP [46] cells or stem cell derived hepatocytes [55].

Another possibility relates to P protein release from RC-DNA. Topoisomerases relax torsional stress by incising DNA [56] via a reversible trans-esterification reaction; an internucleotide phosphodiester bond is opened and a new tyrosyl-DNA-phosphodiester bond to the enzyme is formed, as in RC-DNA. The back-reaction reseals the DNA and releases the topoisomerase in one go. An analogous reaction could “autocatalytically” release P protein and ligate the ends of minus-strand DNA. However, in topoisomerase cleavage complexes reformation of the DNA-DNA phosphodiester bond depends strictly on the proper alignment of the two DNA ends; otherwise the enzyme gets trapped on the DNA [56], with active repair required to resolve the protein-DNA adduct. For RC-DNA a proper alignment of the ends in the terminally redundant minus-strand DNA is difficult to envisage (Figure 3). Together with the absence of viral functions for the other RC-DNA conversion steps this strongly suggests that HBV has to hijack cellular factors for cccDNA formation, and the multifactorial DNA repair system would provide an ample source for all activities required. However, directly tackling such a connection is still challenged by the experimental restrictions in detecting and quantifying HBV cccDNA.

## 5. Human HBV cccDNA—Low Production Versus Difficult Specific Detection

Although this review is largely conceptual a short detour to the bench will serve to highlight some relevant technical issues in cccDNA research. The basic dilemma is that the amounts of human HBV cccDNA in all tractable test systems are low, and that Southern blotting which allows for unambiguous distinction of cccDNA from all other viral DNA forms [19] is an intrinsically insensitive method. 

Infected woodchuck and duck livers may carry ≥50 copies of cccDNA per hepatocyte [57,58]. DHBV-transfected hepatoma cells produce easily Southern blot-detectable amounts of cccDNA. Preventing synthesis of the viral envelope proteins boosts copy numbers to several hundred per cell [36,59,60] by funneling all progeny RC-DNA into the intracellular recycling pathway (Figure 1B) for cccDNA amplification [61]. 

Human HBV cccDNA copy numbers in infected livers appear much lower [62], and may rarely exceed one copy per cell [63]. Also, cccDNA levels in HBV-transfected hepatoma cells are very low [64] although the same cells support high levels of DHBV cccDNA formation [36]; whether distinct features of the viral DNAs, or the viral proteins, or still other factors cause this difference are interesting but unresolved questions. The boost in cccDNA copy numbers by preventing envelope protein production is also much less pronounced for HBV [65], and HBV transgenic mice normally produce no detectable cccDNA at all [66].

More sensitive PCR methods for truly specific cccDNA detection are thus urgently needed but still not available. The main issue is the distinction of cccDNA from the sequence-identical non-cccDNA forms [19] which may vastly outnumber the cccDNA molecules. Primer pairs targeting a genome region that is contiguous only on cccDNA (“over-gap PCR”) can achieve 100- to 1000-fold discrimination [50], and further physical enrichment is possible [67]. However, accurately determining reductions of the anyhow low HBV cccDNA levels, e.g., for evaluation of cccDNA-relevant host factors or anti-cccDNA drugs, remains a challenge [68,69]. Because only cccDNA has no free ends, exonucleases might be used for the selective removal of all non-cccDNA forms. Most widely used [25,62,70] is Plasmid-Safe ATP-dependent DNase (PSD; Epicentre). However, in our hands PSD did not only spare cccDNA from degradation but also DHBV RC-DNA [36], hence the search for alternatives is still ongoing. Data from a model study comparing PSD with the exonucleases from bacteriophages T7 and T5 [71,72] highlight some of the unresolved technical difficulties. 

**Figure 4:**
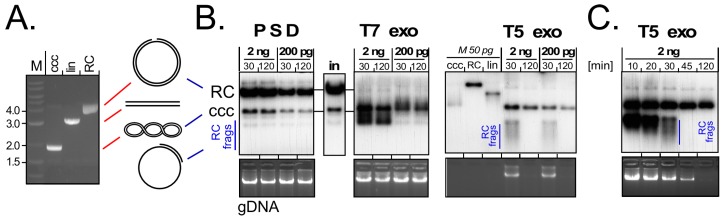
A detour to the bench—suitability of different exonucleases for selective degradation of non-cccDNA forms. (**A**) Model substrates. A 3.2 kb plasmid carrying 800 bp of HBV sequence (lane ccc) was linearized (lane lin) with Bsp QI or nicked (lane RC) with nickase Nt.Bsp QI (both NEB); (**B**) Differential sensitivity towards Plasmid-Safe DNase (PSD) and T7 and T5 exonuclease (T7exo, T5exo). Plasmid RC-DNA and cccDNA were mixed to contain per 50 µL reaction 2 ng or 200 pg of each plasmid DNA plus 4 µg genomic DNA (gDNA) from Huh7 cells (equivalent to 0.6 × 10^6^ cells). After adjusting buffer conditions as recommended by the nuclease manufacturers reactions were supplemented with 10 U of PSD (Epicentre; 1× Plasmid-Safe reaction buffer with 1 mM ATP), or T7 or T5 exonuclease (both NEB; 1× NEBuffer 4) and incubated at 37 °C (PSD, T5 exo) or 25 °C (T7 exo) for 30 or 120 min. After agarose gel electrophoresis gDNA was detected by ethidium bromide staining (bottom panels), HBV plasmid forms by Southern blotting using a ^32^P-labeled HBV DNA probe. M, 50 pg each of the ccc, RC and linear form of the HBV plasmid; (**C**) Detailed time course for T5 exonuclease digestion. An ideal nuclease treatment would completely digest all non-cccDNA forms while fully preserving cccDNA; T5 exonuclease came closest to the first but not to the second criterion.

As substrate we used a 3 kb HBV plasmid either in its ccc form, or in the RC form obtained by treatment with a nickase enzyme (see Figure 4A). Mixtures of two different concentrations of each plasmid form were then mixed with a constant amount of Huh7 cell genomic DNA (gDNA) as carrier and incubated with a defined amount of PSD, or T7 or T5 exonuclease. Aliquots taken after 30 min and 120 min were analyzed by agarose gel electrophoresis and Southern blotting (Figure 4B,C). PSD had no detectable impact at all, i.e., the input pattern of gRNA and of RC- plus cccDNA remained unchanged. T7 exonuclease did not affect the gDNA but led to the rapid disappearance of the RC form while a new band with higher mobility than cccDNA appeared; likely it represents the ssDNA circle remaining after digestion of the linear strand in RC-DNA (see Figure 4A). Most of this material persisted during the 120 min incubation. The most clearcut effects were seen with T5 exonuclease. At 30 min, the gDNA signals were weakened and at 120 min they had disappeared. The RC-DNA signal was no more detectable already at the earliest time point; instead, new fast migrating material (labeled “RC-frags”) was visible after 30 min but no more after 120 min incubation; in line with earlier reports [73] this indicates that T5 (but not T7) exonuclease can attack circular ssDNA. The cccDNA signal persisted, yet its intensity decreased with time. An analysis at shorter intervals (Figure 4C) revealed complete digestion of the fast migrating material upon 45 min incubation; however, at that time also the cccDNA signal was reduced to roughly one half the intensity of the 10 min sample.

In sum, PSD as used here did not generate a pure cccDNA template. More enzyme per DNA, longer incubation times and/or exploiting the higher sensitivity of RC- vs. cccDNA towards heat denaturation may give more favorable results; this also holds for T7 exonuclease. T5 exonuclease came closest to the desired degradation of all non-cccDNA forms but also induced a loss of cccDNA, likely via the endonuclease activity that caused complete degradation of the RC-DNA (Figure 4B,C). There are other potentially useful nucleases, e.g., exonuclease I and exonuclease III from *Escherichia coli* (Hu, J.; unpublished data), but regardless of the specific enzyme it will be mandatory that efforts towards standardized protocols include all enzymatically relevant parameters such as units of enzyme per total amount of substrate DNA, DNA concentration, exact buffer composition, and incubation temperature and duration. 

A recent methodological advance is digital PCR which can give absolute template numbers in a sample without requiring a standard for calibration [74]; however, this does not *per se* increase cccDNA specificity. Notably, even completely noncontiguous HBV DNA fragments can efficiently yield longer, contiguous PCR products via “PCR recombination” [75], underscoring the urgent need for sensitive yet truly specific cccDNA detection.

## 6. Surrogate Models for cccDNA Monitoring

**Figure 5:**
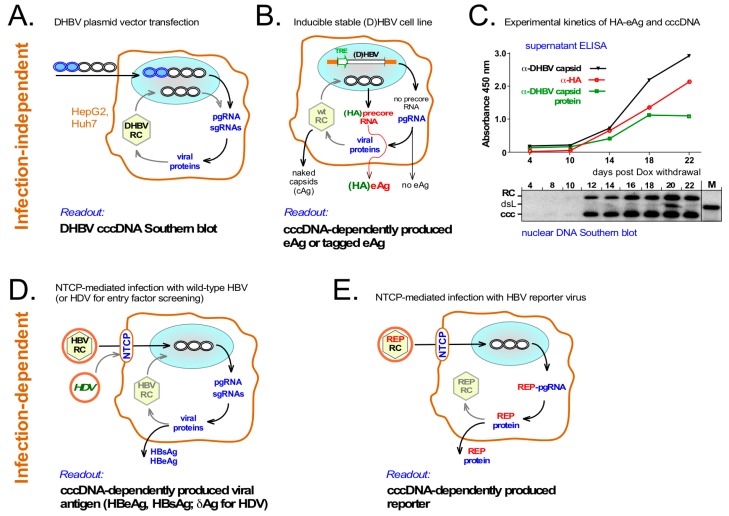
Surrogate models to overcome low production and poor specific detection of HBV cccDNA. (**A**) Transient transfection of DHBV expression vectors into human hepatoma cells. DHBV produces much more cccDNA than HBV in the same human hepatoma cells [36]. Transfected plasmid can be selectively digested using the bacterial methylation-dependent restriction enzyme Dpn I while cccDNA amounts suffice for Southern blot detection; (**B**) Stable, inducibly HBV or DHBV producing hepatoma cell lines. Such cell lines contain a Tet-responsive transactivator (tTA) and an integrated virus expression cassette in which pgRNA is transcribed from an inducible heterologous promoter (e.g., TRE) which does not direct transcription of precore RNA; hence no precore protein or HBeAg is produced. Formation of cccDNA enables precore RNA and precore/HBeAg synthesis. However, specificity of HBeAg detection is limited by crossreactivity with core protein from released naked capsids; this has recently been improved by adding short tags, e.g., HA, specifically to HBeAg; (**C**) Synchronous kinetics of secreted HA-DHBeAg and cccDNA production in an inducible DHBV HepG2 line encoding HA-tagged DHBeAg. Expression of pgRNA was induced by Dox withdrawal; at the indicated time points intact DHBV capsids, DHBV capsid protein (as present in DHBeAg and disassembled capsids) and HA-tag in the culture supernatants were monitored by ELISA; in parallel, nuclear DNAs were analyzed by Southern blotting; (**D**) Infection-dependent cccDNA formation with wild-type HBV. Productive infection of NTCP-expressing cells depends on prior cccDNA formation, resulting in generation of viral antigens; hence HBsAg and HBeAg can serve as surrogate markers for cccDNA production. Interference with other infection steps would cause the same readout; entry-specific factors may be identified by using HDV [17] which shares only the early infection steps with HBV; (**E**) Improved detection of infection via HBV reporter vectors. Easily detectable reporters (REP) encoded by modified HBVs and expressed in a cccDNA-dependent fashion would allow more sensitive and better quantifiable monitoring. As yet, however, such HBV vectors are much less advanced than for other virus families.

In view of the problems with cccDNA quantitation several cell culture systems could provide useful workarounds, as summarized in Figure 5. Ongoing improvements may make these surrogate models suitable for high-throughput screening towards identifying cccDNA-relevant host factors and/or chemical inhibitors of cccDNA formation.

### 6.1. Infection-Independent cccDNA Model Systems

The high production of cccDNA by DHBV even in human hepatoma cells [36] greatly facilitates its clearcut detection by Southern blotting (see Figure 5A). This direct readout for the impact of inhibiting host factors on RC-DNA to cccDNA conversion was used in the identification of tyrosyl-DNA-phosphodiesterase 2 (TDP2) as a host DNA repair enzyme that can release P protein from RC-DNA [49]. However, Southern blotting is not suited for higher throughput applications, and despite the overall similarity between DHBV and HBV RC-DNA, there may be differences as to which sets of host factors are optimal for their conversion into cccDNA [76]. 

The second kind of systems (see Figure 5B) relies on stable, inducibly HBV (or DHBV) producing cell lines such as the TetOFF HBV lines HepAD38 [77] and HepG.117 [64]. HBV pgRNA is transcribed from a chromosomally integrated cassette under control of a tetracycline (Tet) response element (TRE) promoter and a Tet-repressor based trans-activator (tTA) that binds the TRE promoter only in the absence of Tet. Tet withdrawal induces transcription from the TRE promoter of pgRNA but not precore RNA (see Figure 1) whose start site lies about 30 nt upstream. From pgRNA a first round synthesis of RC-DNA containing nucleocapsids is initiated which may then establish nuclear cccDNA. If so, this allows precore RNA and thus HBeAg production. Hence HBeAg can serve as a surrogate marker for cccDNA formation, and two reportedly specific small compound cccDNA inhibitors were identified in this way [78]. 

However, discrimination of HBeAg from the cccDNA-independently produced core protein is problematic owing to their largely identical amino acid sequences; furthermore, not only HBeAg but also non-enveloped capsids are found in the culture supernatant [79]; this holds also for DHBV (Dörnbrack, K.; Costa, C.; Nassal, M.; unpublished data). To overcome this problem, others [80] and we (Dörnbrack, K.; Costa, C.; Verrier, E.; Nassal, M; unpublished data) have engineered coding sequences for the small hemagglutinine (HA) tag into the precore regions of HBV and/or DHBV such that only HBeAg becomes HA-tagged and the negative impact on replication via the precore-overlapping ε sequence remains limited. Figure 5C shows the accumulation of nuclear cccDNA (and RC-DNA) in a TetOFF HepG2 line producing HA-tagged DHBeAg. Southern blot signals emerged at day 12 post induction and concomitantly ELISA signals became detectable in the culture supernatant for intact DHBV capsids (black line), DHBV capsid protein (green line; DHBeAg or disassembled capsids) and, most importantly, for HA (red line). The desired presence of the HA tag on DHBeAg was proven by the distinct size of this protein on Western blots (data not shown). 

Hence, these and similar stable cell lines should become very useful tools for the screening of host factors involved in the intracellular steps of hepadnaviral replication, including cccDNA formation. 

A complementary new tool is provided by the minicircle technology which allows to produce cccDNA-like molecules (just containing a short bacterial recombination site but no plasmid backbone) in *E. coli*. When transfected into hepatoma cells, these molecules resemble true cccDNA much more than conventional plasmid vectors [81], and when combined with an integrated reporter, they allow to monitor transcriptional activity of the artificial cccDNA-like molecule and its regulation [82]. Obviously, though, de novo biogenesis of cccDNA cannot be investigated.

### 6.2. Infection-Dependent Systems

Productive HBV infection clearly depends on cccDNA formation but cell culture infection systems were restricted, until recently, to primary human [83] or tupaia hepatocytes [84,85], chimeric mice with humanized liver [86], or a bipotent liver progenitor cell line, HepaRG [87], which can be differentiated into hepatocyte-like cells susceptible to HBV infection. In these cells the strict dependence of infection on HBx previously only seen in vivo [30,48,88,89] was reproduced [27]; furthermore, it was shown that HBx does not affect cccDNA formation as such but rather is required for cccDNA transcriptional activity. However, proper HepaRG differentiation requires a lengthy and elaborate procedure [90].

Experimental flexibility was thus greatly expanded by the discovery of the bile acid transporter sodium taurocholate cotransporting polypeptide (NTCP) as a receptor for HBV and its satellite hepatitis D virus (HDV), which exploits HBV’s envelope to enter new host cells [15,16]. Stable expression of NTCP makes HepG2 cells susceptible to HBV infection, providing now a relatively robust model to investigate cccDNA formation under controlled conditions (see Figure 5D).

Notably though, reasonable infection rates require multiplicities of infection (MOIs) in the range of 1000 viral genome equivalents or more per cell plus additives such as dimethyl sulfoxide (DMSO) and polyethylene glycol [91]. Although recent RNA interference (RNAi) screens targeting a limited number of host factors have already uncovered Glypican 5 (GPC5; [17]) and DNA polymerase kappa (POLK [46]; see below) as new HBV dependency factors, higher throughput applications will require further improvements [92].

One approach is finding more efficiently infectable cells. For HCV some sublines of the principally infectable Huh7 cell line exerted much higher susceptibility [93], and this may also hold for HBV infection of NTCP-HepG2 cell clones. Another option are different cell types; for instance, promising infection results were recently obtained with stem cell-derived hepatocyte-like cells [55,94]. 

Alternatively, the sensitivity of infection detection may be enhanced by engineered HBV reporter viruses encoding easily traceable (e.g., fluorescent or bioluminescent) molecules which are expressed as a result of productive infection (see Figure 5E). Such reporter viruses have been instrumental for better understanding the life-cycles of various virus families as well as for anti-virals development [95]. For HBV, however, the compact organization of its genome imposes massive constraints on any sequence manipulation. Since earlier attempts [96] some progress has been made [97,98], but most HBV vectors so far suffer from strongly reduced replication capacity [98] and/or genetic instability of the recombinant genomes when their size exceeds that of the natural virus (Sun, D.; Gonzalez, M.M.; Nassal, M.; unpublished data). However, given the enormous potential of HBV reporter viruses, putting more efforts into further improved, innovative vector designs is certainly highly worthwhile. 

## 7. Evidence for a Connection between HBV and the Host DNA Damage Response

There is no evidence that HBV could perform the multiple steps of RC- to cccDNA conversion without exploiting host nucleic acid manipulating enzymes, the richest source for which is the cell’s DNA damage response (DDR) system. Several additional lines of evidence support such an interaction. One is the accumulating evidence that all viruses with a nuclear phase, and likely even RNA viruses with a purely cytoplasmic replication cycle [99], have to cope with the DDR [100,101,102], on the fundamental level of viral replication (which can be promoted or impaired) and host innate defenses [103]. Key to these multipronged effects is that the DDR comprises a whole network of pathways that sense, signal and repair DNA lesions and in the process profoundly affect the cell cycle; this can include induction of cell death if damage is beyond repair to ensure survival on the organismal level. Hence, different viruses have to cope in different ways with the DDR; some deliberately induce it (“exploit”) while others actively prevent it (”avoid”), and HBV is likely no exception. Further hints for an HBV-DDR interaction come from the frequent integration of HBV sequences in HCC, circularization of HBV dsL-DNA, and the occasional identification of DDR components in interaction screens, mainly with the HBx protein. 

### 7.1. The Host DDR—A Simplified Overview

DNA damage describes any of a huge variety of deviations from a perfectly double-stranded DNA structure, including chemically and/or radiation-induced base-modifications, intra- and interstrand crosslinks, mismatches, abasic sites and/or covalent adducts of small or proteinaceous moieties. Each cell in our body may experience 10,000 or more such damage events per day [104]. As all of these lesions can interfere with proper replication and transcription, all cells are equipped with sophisticated DNA repair systems that ensure genome integrity. 

**Figure 6:**
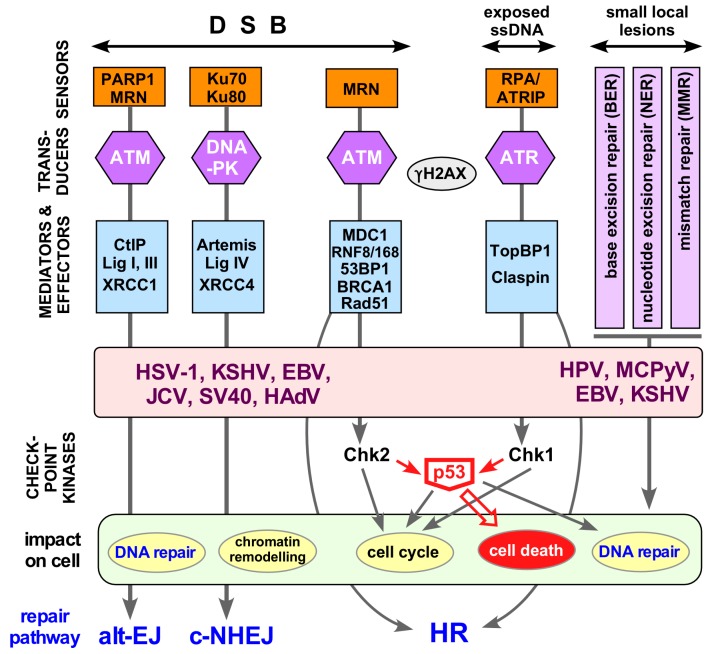
The host DNA damage repair response and viral interference. Double-strand DNA breaks (DSBs), exposed single stranded DNA (ssDNA) at collapsed replication forks and various kinds of local DNA lesions are detected by sensors that mark the site of damage. Transducers, and mediator plus effector proteins transmit and amplify the signal throughout the cell, resulting in a huge influx of factors to repair damage and remodel chromatin. The key apical transducer kinases are ATM (Ataxia telangiectasia mutated), ATR (Ataxia telangiectasia and Rad3-related), and DNA-PK (DNA-dependent protein kinase). These Ser/Thr kinases regulate DNA replication, DNA repair, cell-cycle checkpoint control (e.g., via Chk1, Chk2), and if necessary cell death (e.g., via p53) by recruitment of specific effector proteins. A hallmark of the DNA damage respone (DDR) is phosphorylation of histone H2AX to generate γH2AX, and the formation of large γH2AX foci around the site of damage. c-NHEJ and alt-EJ are error-prone and always active; high-fidelity repair via homologous recombination (HR) requires an intact sister chromatide as template and thus is largely restricted to the S and G2 phases of the cell cycle. More and more viruses are known to exhibit a complex relationship with the DDR [101,106] to maintain efficient viral replication and onset of gene expression. Only some well established DNA virus examples are shown (HSV-1, herpes simplex virus type 1; EBV, Epstein-Barr-virus; KSHV; Kaposi’s sarcoma herpesvirus; JCV, JC virus; SV40, simian virus 40; HPV, human papillomavirus; MCPyV; Merkel cell polyomavirus; HAdV, human adenovirus) but RNA viruses are joining the list [99]. See text for further details. Abbreviations: 53BP1, P53-binding protein 1; BRCA1, Breast cancer susceptibility gene 1; Chk, Checkpoint kinase; CtIP, C-terminal interacting protein; DSB, double-strand DNA break; MDC1, Mediator of DNA damage checkpoint; PARP1, poly ADP ribose polymerase 1; RNF, Ring finger protein; TopBP1, topoisomerase-IIbeta-binding protein 1; XRCC, X-ray repair complementing defective repair in Chinese hamster cells 1.

The diversity of DNA damage events calls for a matching diversity of damage recognition mechanisms (for a comprehensive overview see [105]) and repair activities which are embedded into a much larger network that integrates the responses to DNA lesions in a coordinated, spatiotemporally controlled way. A highly simplified scheme of this network is shown in Figure 6.

Small local DNA lesions, including base modifications or mismatches from DNA replication, are repaired by base excision repair (BER), nucleotide excision repair (NER) or mismatch repair (MMR). Thereby the damaged site is excised from the DNA, often together with a few neighboring residues, followed by gap repair synthesis and strand ligation, using the undamaged strand as template (right part in Figure 6).

The most detrimental lesions are strand breaks. Double-strand DNA breaks (DSBs) usually induce an immediate DDR. Single-strand DNA breaks (SSBs) and exposed ssDNA regions are obligatory intermediates in nearly all nucleolytic repair pathways. The major break repair mechanisms are the error-prone classical nonhomologous end-joining (c-NHEJ) and alternative EJ (alt-EJ; left part in Figure 6). A further, more recently defined mechanism, single-strand annealing SSA (not shown), joins interspersed repeats with deletion of the in-between sequences [107]). The alternative is homologous recombination (HR), a high fidelity repair mechanism requiring a homologous repair template, usually in the form of a sister chromatide. 

The key damage sensors in NHEJ are Ku70/Ku80 (c-NHEJ) and PARP1 (alt-EJ), which recruit DNA-activated protein kinase (DNA-PK; [108]) or the trimeric MRN complex, respectively, consisting of the nuclease MRE11, Nijmegen breakage syndrome 1 (Nbs1) and the ATPase Rad50, a Smc family member. This ultimately results in the recruitment of DNA ligase IV (c-NHEJ) or DNA ligases I and III (alt-NHEJ) and rejoining of the DNA ends. 

HR-mediated repair is embedded into a complex signaling network that couples DNA repair to the cell cycle [109]. Halting the cycle allows time for repair whereas too extensive damage usually induces cell death so as to prevent cancer. The respective signaling cascades are initiated by either of the two major transducer kinases [110] Ataxia telangiectasia mutated (ATM), or Ataxia telangiectasia and Rad3 related (ATR), like DNA-PKcs members of the phosphatidylinositol 3-kinase-related kinase (PI3KKs) family. 

DSBs destined for HR-repair are recognized by the MRN complex which also has nuclease and signaling roles through autophosphorylation and phosphorylation of downstream targets including ATM [111,112]. Phospho-ATM phosphorylates further downstream effectors such as the histone variant H2AX. Phosphorylated “γH2AX” leads to the coordinated accumulation of more MRN and ATM and the adaptor MDC1 at the site of damage [113], amplification of γH2AX and activation of the cell cycle checkpoint kinase CHK2, which in turn phosphorylates and thereby stabilizes p53. CHK2 also phosphorylates the phosphatase CDC25C, preventing activation of cyclin-dependent kinase 1 (CDK1), with concomitant G2 arrest. Exposed ssDNA and SSBs are bound by replication protein A (RPA), inducing recruitment of factors activating the second major signaling kinase, ATR [114,115] which promotes cell cycle regulation through CHK1, eventually inhibiting CDK1 and CDK2. 

Cell-death as a result of non-repairable DNA damage, via apoptosis or necroptosis (a regulated form of necrosis involving, in contrast to apoptosis, spill-out of intracellular contents to the extracellular space), is largely induced through p53 [116], both via transcription of proapoptotic genes and by affecting mitochondrial outer membrane permeabilization.

### 7.2. Other Viruses and the DDR

From the viral viewpoint the DDR resembles a self-service store for repair factors; tapping this reservoir is smart yet also risky, owing to the emerging coupling of DNA damage sensing to innate immunity [103,117]. From the cell’s viewpoint, too careless a virus provides an opportunity for the DNA repair system to interfere with virus propagation, if necessary by killing the infected cell. Not surprisingly then, many viruses undergo multifaceted interactions with the DNA repair system (Figure 6). The two major strategies are to usurp beneficial aspects of DNA repair, or to block its detrimental aspects. However, a strict categorization in “exploit or avoid” is an oversimplification as the needs and risks for the same virus may differ at different stages of its replication cycle. 

Given the exquisite sensitivity with which cells can detect subtle alterations in their DNA (in the range of 1 part per billion) it is not that surprising that viral genomes in the nucleus do not go undetected. DDR triggers can be unusual genome structures as such [100,101,102], e.g., ssDNA in parvoviruses [118], linear dsDNA as in adenoviruses, yet also replication intermediates, e.g., linear retrovirus DNA prior to integration; HBV replicative intermediates would most likely match this category. Other triggers are viral proteins that interact incidentally with host DNA, such as the HPV E1 helicase, or directly target DDR components to manipulate their functions, as now even seen for RNA viruses with an exclusively cytoplasmic life-style [99]. It is also obvious that some DDR aspects may be beneficial for one virus yet detrimental to another; furthermore the specific requirements a virus has may change during its replication cycle. For instance, viruses infecting quiescent cells may often opt to activate cell cycle progression into S phase, and most viruses will tend to block premature p53-mediated cell death. Hence, an emerging theme is that viruses exploit selected beneficial aspects of the DDR, while avoiding untoward downstream consequences; this may involve certain differences between viral and cellular DDR induction [119,120].

Several recent reviews comprehensively summarize how individual viruses cope with the cellular DDR [100,101,102,106,121,122,123,124,125,126]. The diversity of these interactions underlines how important the DDR is as a host-virus interface. An extra boost to this concept comes from the recent identification of key DDR components, such as Ku70/Ku80, DNA-PK, MRE11 and RAD50, as sensors of foreign, including viral, DNA which induce a type-I IFN response, mostly via the stimulator of IFN genes/cyclic GMP–AMP synthase (STING/cGAS) axis [103]. Not surprisingly, viral counter-strategies are already being identified [117,127]. For HBV, however, most current evidence for an interaction with the DDR is indirect.

### 7.3. Crosstalk between HBV and DNA Repair—Integration and Viral dsL-DNA Circularization

Most HBV-related HCCs contain integrated viral DNA [10], usually at random genomic sites [128]. However, integration occurs long before cancer becomes manifest [129,130], and is also seen with the noncancerogenic DHBV [131]. Hence integration appears to be a common consequence of hepadnavirus infection; as hepadnaviral genomes lack an integrase-like open reading frame integration must be performed by cellular activities.

Recent deep sequencing data confirmed the random nature of genomic integration sites [132], but the viral DNA breakpoints show a clear bias for the region around the 3′ end of the minus-strand. Notably, HBV reverse transcription commonly yields a small proportion of double-stranded linear (dsL) DNA where RNA-primed plus-strand DNA synthesis occurred without the template switch required for circularization [7]. Hence dsL-DNA presents itself to the cell like DNA with a double-strand break (DSB), except for the non-DNA 5′ ends (see Figure 6). Because DSBs are the most dangerous DNA lesions it is likely that the free DNA ends in dsL-DNA represent a potent trigger for a DDR, and integration is one way of resolving this issue. 

An alternative is dsL-DNA circularization, which was directly demonstrated by the formation of cccDNA-like molecules from DHBV genomes with engineered defects in RC-DNA formation [133,134]. This strongly resembles freshly synthesized (linear) retroviral DNA that can either integrate (as desired by the virus) or be “repaired” to dead-end single long terminal repeat (LTR) circles via intramolecular homologous recombination of the two LTRs, and double LTR circles by non-homologous end-joining (NHEJ) [47,135].

**Figure 7:**
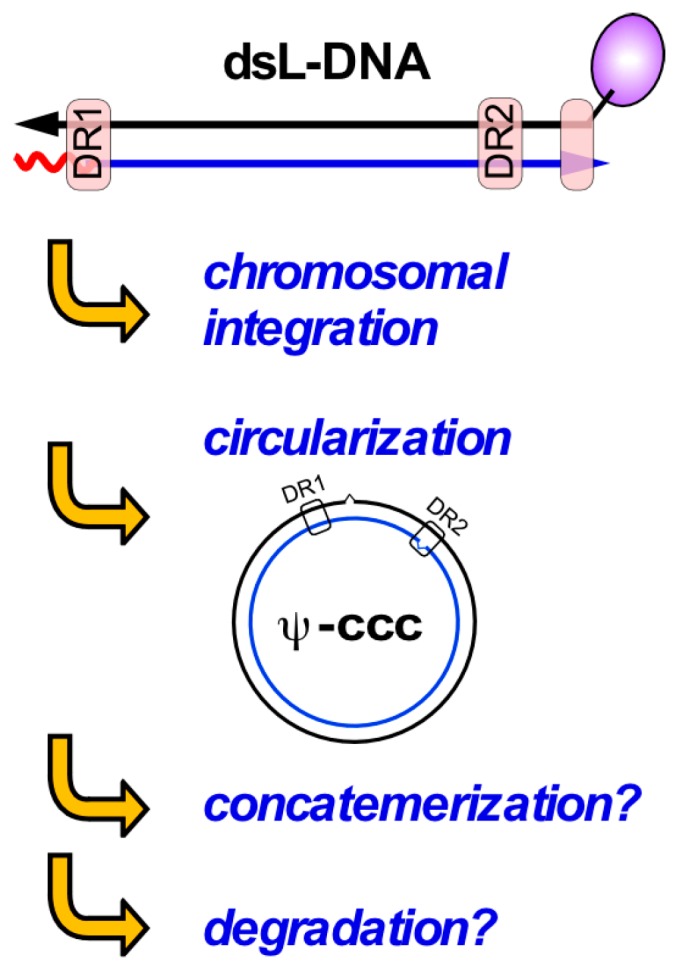
Double-strand linear (dsL) DNA—a free-ended double-strand DNA break mimic? Hepadnaviral dsL-DNA is a common replication by-product from in-situ priming of plus-strand DNA. Like other linear DNAs dsL-DNA likely evokes repair responses that remove the free ends, e.g., by chromosomal integration, circularization (as for retroviral 2LTR-circles), concatemerization and/or exonucleolytic degradation. Circularization appears to occur mainly via error-prone non-homologous end-joining, yielding often defective cccDNA-like molecules (Ψ-cccDNA). Concatemerization and degradation via exonucleases have not explicitly been demonstrated.

That also hepadnaviral dsL-DNA is circularized by NHEJ [133,134] is supported by the involvement of Ku80 [136], a typical component of the c-NHEJ pathway (see Figure 6). It should be emphasized that c-NHEJ and alt-EJ (also known as microhomology-mediated EJ) are error-prone pathways [137] often causing small insertions and deletions (indels); this is exploited by current genome-editing knock-out techniques. As all nt of the hepadnaviral genome have coding function any indel will be deleterious; moreover, dsL-DNA bears the small “r” redundancy which is unlikely to be accurately removed. Hence, circularization of dsL-DNA is not an effective alternative to the normal RC- to cccDNA pathway; to indicate this fact, the circular DNA shown in Figure 7 is termed Ψ-cccDNA. Notably, transfected linear unit-length HBV DNA (excised from an appropriate plasmid) can also give rise to circular molecules [26] but in the absence of the typical 5′- end modifications of viral DNA the pathways may not be exactly the same.

A related strategy for minimizing the number of free DNA ends is concatemerization. A prime example for this type of damage response are the linear (about 36 kb) dsDNA genomes of adenoviruses which, unless counteracted by the virus, are “repaired” into concatemers too large to be packaged into the viral capsids [138]. To prevent this unproductive repair, adenoviruses have evolved countermeasures that actively inhibit key DDR factors involved in DSB recognition and repair, such as the MRN complex [106]. Whether hepadnaviral dsL-DNA can be concatemerized is not known; in the absence of hepadnaviral replication factories with high local genome concentration, circularization may be the preferred reaction. Lastly, the free ends in dsL-DNA may represent targets for exonucleolytic degradation, but this has not yet been investigated. 

In sum, these considerations strongly support an interaction of hepadnaviruses with cellular DNA repair, with the free ends of dsL-DNA as a likely major trigger that is shut off by integration and circularization, and perhaps by concatemerization and degradation. While RC-DNA bears less resemblance to broken DNA, its 5’ terminal protein and RNA modifications are still obvious tags for distinction from normal DNA (see below). Hence, both forms of hepadnavirus DNA could conceivably contribute to DDR activation.

### 7.4. Does HBx Connect HBV to the Host DDR?

A connection to DNA repair was surmised early-on based on the association of HCC with chronic hepatitis B and the known correlation of cancer with improper DNA damage repair and/or failure in preventing cells with damaged DNA to proliferate. Various aspects of HBV expression were reported to impact on DNA repair [139,140,141,142,143,144,145], but most attention was paid to HBx, owing to its suspected role as an oncogene. Notably, HBx is now rather seen as a cofactor enhancing the transforming activity of true carcinogens [29,146,147]. 

Among the dozens of reported HBx interacting proteins [148] there were also DNA repair proteins [149,150,151,152], including p53 [153], the “guardian of the genome”, and UV-damaged DNA binding protein 1 (DDB1), first identified in 1995 as X-associated protein 1 (XAP-1) [154]. DDB1 is one of few repeatedly confirmed HBx interactors [28], culminating in the discovery that the HBx-DDB1 interaction mediates degradation of the Smc5/6 complex [31,32,33,34] which binds DNA and has as yet not well defined roles in DNA repair [34,155]. 

**Figure 8:**
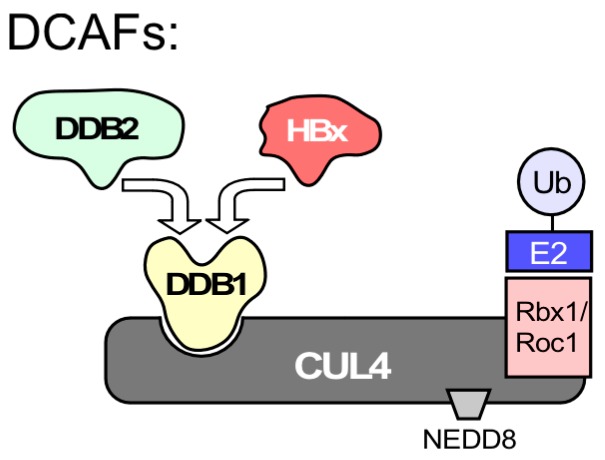
HBx-DDB1 interaction does not necessarily indicate a direct connection to DNA repair. UV-damaged DNA binding protein 1 (DDB1) was reported early on as an HBx interactor. However, different from what the name implies DDB1 does not directly bind to damaged DNA; this function is taken by the distinct DDB2 protein. DDB1’s major function is that of an adaptor in the E3 ubiquitin ligase CRL4, comprising the cullin 4 (CUL4) scaffold protein, a RING finger domain protein (Rbx1 or Roc1) which mediates binding of a ubiquitin conjugating E2 enzyme, and a regulatory site for modification with NEDD8. For ubiquitylation of specific target proteins, DDB1 must interact with DDB1-CUL4 associated factors (DCAFs) which act as substrate receptors. DDB2 is one of multiple cellular DCAFs while HBx is a viral DCAF [31,32,33]. As HBx binds to the same site on DDB1 as cellular DCAFs do [162] their binding is mutually exclusive.

However, binding of HBx to DDB1 does not by itself establish a connection to DNA damage. Though also involved in DNA repair, DDB1 does not directly bind to damaged DNA; this function is rather due to DDB2 [156,157], one of various DDB1 partner proteins. DDB1 acts largely as an adaptor in Cullin4 RING E3 ubiquitin ligases (CRL4s), multisubunit complexes that mediate ubiquitinylation of substrate proteins (Figure 8), marking them for proteasomal degradation [158]. Target recognition requires additional substrate receptors; for CRL4 these are collectively termed DDB1- and CUL4-associated factors (DCAFs), one of which is DDB2. Notably DCAF1 is also known as Vpr binding protein; binding of the HIV-1 accessory protein Vpr to DCAF1 mediates degradation of repair factors such as helicase-like transcription factor (HLTF) and uracil DNA glycosylase (UNG2) [159,160]; similarly, HIV-2 Vpx degrades the host restriction factor SAMHD1 [161].

Based on structural data [162], HBx acts as a viral DCAF for DDB1 and binds to the same site on DDB1 as host DCAFs; hence DDB1 can bind either HBx or DDB2 but not both. Hence rather than on direct binding to damaged DNA an HBx-mediated HBV linkage to DNA repair could be based on Smc5/6 degradation yet also on other, additional cellular HBx targets [163].

### 7.5. HBV RC-DNA to cccDNA Conversion—A Direct Case for Host DNA Repair Dependency

**Figure 9:**
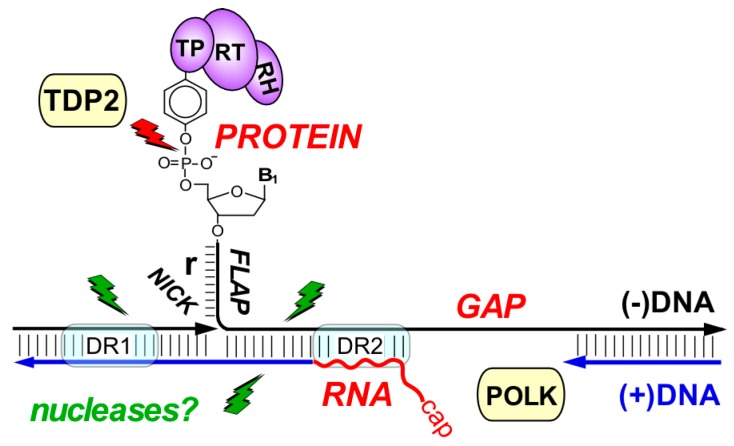
Summary of hepadnaviral RC-DNA as a multi-target DNA repair substrate. Close-up of the molecular peculiarities in RC-DNA. The unusual features of RC-DNA concentrate on a small region encompassing direct repeats DR1 and DR2. P protein is linked to the 5′ end of minus-strand DNA through a tyrosyl-DNA phosphodiester bond; the small “r” redundancy forms a flap preceded by a nick. The incomplete plus-strand starts with RNA and leaves a gap that makes the opposite minus-strand single-stranded. The host enzyme TDP2 can cleave the tyrosyl-DNA-phosphodiester bond to release P protein [49] yet as in repair of cellular protein-DNA adducts alternative, probably nucleolytic repair pathways are likely to exist (green lightning symbols). Translesion DNA polymerase κ (POLK) is thought to fill-in the gap in plus-strand DNA [46] but can probably also be substituted for by other repair polymerases. Factors required for the other RC-DNA modifications have not yet been identified.

The most immediate benefit of the host DNA repair system for HBV would be the provision of enzymatic activities that help converting RC-DNA into cccDNA [19]. As summarized in Figure 9, hepadnaviral RC-DNA contains many unusual molecular features that are not present in normal cellular DNA, except as temporary intermediates or improper side-products of DNA metabolism that evoke a DDR. Recent data have indeed provided evidence for the involvement of DNA repair factors in RC-DNA to cccDNA conversion. The first study was based on the chemical similarity of the P protein linkage to minus-strand DNA through a 5′-tyrosyl-DNA-phosphodiester bond, which also occurs in trapped cellular topoisomerase cleavage complexes. These are either repaired by specific tyrosyl-DNA-phosphodiesterases (TDP1, TDP2; [164]), or by one of various nucleolytic pathways [165], whereby the trapped protein is excised together with a piece of DNA (green lightning symbols in Figure 9). TDP2, though not TDP1, was able to release P protein from HBV and DHBV RC-DNA in vitro, and RNA interference-mediated depletion of TDP2 from human hepatoma cells significantly slowed down DHBV RC- to cccDNA conversion [49]; however, cccDNA formation was not ablated even upon TDP2 knock-out [76]. By analogy to the weak phenotypes caused by TDP knockouts in yeast this most likely reflects that other, likely nucleolytic, repair pathways can step-in. Conceivably, pathway choice may also be affected by subtle differences in DHBV vs. HBV RC-DNA (or processing intermediates); hence directly comparing the two viral DNAs in the same human cell background, including in the absence vs. presence of the other virus’ proteins, will certainly be highly worthwhile. 

A second recently identified host enzyme important for RC- to cccDNA conversion is DNA polymerase kappa (POLK), one of the Y-family translesion DNA polymerases [166,167] that can by-pass damaged nucleotides in stalled replication forks; whether this ability is essential here to fill-in the gap in plus-strand DNA is not yet clear. Knock-down, knock-out and pharmacological inhibition of POLK all reduced cccDNA production but also not to zero, probably again reflecting redundancy in the repair pathways; depletion of DNA polymerases eta (POLH) and lambda (POLL) also had some negative impact on cccDNA formation. However, which step(s) POLK and possibly POLH and POLL exactly perform in cccDNA formation remains to be determined. The latest addition, also found via an RNAi screen, is pre-mRNA processing factor 31 (PRPF31; [168]. As PRPF31 is normally involved in splicing, further data will be required to assess how this factor could enhance cccDNA formation.

Altogether, it is likely that the new cccDNA-dependent test systems (see Figure 5) will provide the means to identify ever more of the host factors involved in RC- to cccDNA conversion. This should also turn up non-enzymatic factors belonging to the complex DDR network as a whole. Potential therapeutic implications of such data are covered in several recent reviews [19,169,170,171].

## 8. Conclusions and Open Questions

Understanding the molecular details of RC-DNA conversion into the cccDNA minichromosome will be a critical asset in developing strategies for a cure of chronic hepatitis B. There are still technical obstacles hampering high-throughput approaches for the global identification of host factors involved in the process, yet several lines of evidence support a crucial role for the cellular DDR; however, at present many options are open as to how such an interaction may manifest itself for HBV. Hepadnaviral genomes are amongst the smallest animal virus genomes known, implying a particularly strong dependence on host factors, including on the DNA damage repair machinery. Conversely, they also lack the sophisticated genetic equipment that larger viruses invest in handling the challenges of a close encounter with the DDR. As outlined above, RC-DNA appears to present a sufficient number of unique molecular features to make itself conspicuous to cellular DNA surveillance and induce the respective repair activities. However, which are the cellular sensors and which of the different programs are activated, if any? With HBV infecting largely quiescent hepatocytes, does this depend on the cell cycle? Could the seemingly useless fraction of dsL-DNA have a DDR-triggering role that facilitates cccDNA formation? Typical for cellular DNA damage repair is the megabase-wide spreading of γH2AX around the break site; as recently shown, even the 36 kb adenovirus genomes are too short for this [119]. How then does the tiny HBV genome behave compared to host genomic DNA?

Would DDR induction also induce an innate response that jeopardizes the virus [172]? Two recent studies in HepaRG cells concluded that HBV actively suppresses innate responses, possibly via its P protein [173,174]; however, other studies suggest that HBV is a stealth virus that simply goes undetected and hence can abstain from actively counteracting such immune responses [33].

Not the least, there should be differences in the cell’s perception of RC-DNA that has freshly been released from the nucleocapsid and is just ready to be converted into cccDNA versus long-term chromatinized cccDNA. Is cccDNA as a perfectly double-stranded circle without free ends protected from cellular damage surveillance? Is the HBV minichromosome a safe store for cccDNA as it is inconspicuouly similar to cellular chromatin? Or does its small size and possible association with viral proteins cause it to be recognized? If so how does HBV prevent a permanent DDR activation and/or apoptosis?

Questions like these had often tried to be addressed using the coarse experimental systems, such as transient overexpression of individual gene products, that until recently dominated the field. With further improvements, the new cell culture systems promise more physiological answers, and these will also impact on the chances for developing new curative treatments of chronic hepatitis B.

## References

[1] Trepo C., Chan H.L., Lok A. (2014). Hepatitis B virus infection. Lancet.

[2] Stanaway J.D., Flaxman A.D., Naghavi M., Fitzmaurice C., Vos T., Abubakar I., Abu-Raddad L.J., Assadi R., Bhala N., Cowie B. (2016). The global burden of viral hepatitis from 1990 to 2013: Findings from the Global Burden of Disease Study 2013. Lancet.

[3] WHO (2017). Fact sheet 297 Cancer 2017.

[4] Götte M., Feld J.J. (2016). Direct-acting antiviral agents for hepatitis C: Structural and mechanistic insights. Nat. Rev. Gastroenterol. Hepatol..

[5] Moradpour D., Grakoui A., Manns M.P. (2016). Future landscape of hepatitis C research—Basic, translational and clinical perspectives. J. Hepatol..

[6] Beck J., Nassal M. (2007). Hepatitis B virus replication. World J. Gastroenterol..

[7] Nassal M. (2008). Hepatitis B viruses: Reverse transcription a different way. Virus Res..

[8] Seeger C., Mason W.S. (2015). Molecular biology of hepatitis B virus infection. Virology.

[9] Lieberman P.M. (2016). Epigenetics and Genetics of Viral Latency. Cell. Host Microbe.

[10] Tu T., Budzinska M.A., Shackel N.A., Urban S. (2017). HBV DNA Integration: Molecular mechanisms and clinical implications. Viruses.

[11] Isorce N., Lucifora J., Zoulim F., Durantel D. (2015). Immune-modulators to combat hepatitis B virus infection: From IFN-alpha to novel investigational immunotherapeutic strategies. Antiviral Res..

[12] Gish R., Jia J.D., Locarnini S., Zoulim F. (2012). Selection of chronic hepatitis B therapy with high barrier to resistance. Lancet Infect. Dis..

[13] Rehermann B., Ferrari C., Pasquinelli C., Chisari F.V. (1996). The hepatitis B virus persists for decades after patients’ recovery from acute viral hepatitis despite active maintenance of a cytotoxic T-lymphocyte response. Nat. Med..

[14] Seto W.K., Chan T.S., Hwang Y.Y., Wong D.K., Fung J., Liu K.S., Gill H., Lam Y.F., Lie A.K., Lai C.L. (2014). Hepatitis B reactivation in patients with previous hepatitis B virus exposure undergoing rituximab-containing chemotherapy for lymphoma: A prospective study. J. Clin. Oncol..

[15] Ni Y., Lempp F.A., Mehrle S., Nkongolo S., Kaufman C., Falth M., Stindt J., Königer C., Nassal M., Kubitz R. (2014). Hepatitis B and D viruses exploit sodium taurocholate co-transporting polypeptide for species-specific entry into hepatocytes. Gastroenterology.

[16] Yan H., Zhong G., Xu G., He W., Jing Z., Gao Z., Huang Y., Qi Y., Peng B., Wang H. (2012). Sodium taurocholate cotransporting polypeptide is a functional receptor for human hepatitis B and D virus. eLife.

[17] Verrier E.R., Colpitts C.C., Bach C., Heydmann L., Weiss A., Renaud M., Durand S.C., Habersetzer F., Durantel D., Abou-Jaoude G. (2016). A targeted functional RNA interference screen uncovers glypican 5 as an entry factor for hepatitis B and D viruses. Hepatology.

[18] Gallucci L., Kann M. (2017). Nuclear Import of Hepatitis B Virus Capsids and Genome. Viruses.

[19] Nassal M. (2015). HBV cccDNA: Viral persistence reservoir and key obstacle for a cure of chronic hepatitis B. Gut.

[20] Chevaliez S., Hezode C., Bahrami S., Grare M., Pawlotsky J.M. (2013). Long-term hepatitis B surface antigen (HBsAg) kinetics during nucleoside/nucleotide analogue therapy: Finite treatment duration unlikely. J. Hepatol..

[21] Lai C.L., Wong D., Ip P., Kopaniszen M., Seto W.K., Fung J., Huang F.Y., Lee B., Cullaro G., Chong C.K. (2017). Reduction of covalently closed circular DNA with long-term nucleos(t)ide analogue treatment in chronic hepatitis B. J. Hepatol..

[22] Bock C.T., Schranz P., Schroder C.H., Zentgraf H. (1994). Hepatitis B virus genome is organized into nucleosomes in the nucleus of the infected cell. Virus Genes.

[23] Bock C.T., Schwinn S., Locarnini S., Fyfe J., Manns M.P., Trautwein C., Zentgraf H. (2001). Structural organization of the hepatitis B virus minichromosome. J. Mol. Biol..

[24] Newbold J.E., Xin H., Tencza M., Sherman G., Dean J., Bowden S., Locarnini S. (1995). The covalently closed duplex form of the hepadnavirus genome exists in situ as a heterogeneous population of viral minichromosomes. J. Virol..

[25] Lucifora J., Xia Y., Reisinger F., Zhang K., Stadler D., Cheng X., Sprinzl M.F., Koppensteiner H., Makowska Z., Volz T. (2014). Specific and nonhepatotoxic degradation of nuclear hepatitis B virus cccDNA. Science.

[26] Belloni L., Pollicino T., De Nicola F., Guerrieri F., Raffa G., Fanciulli M., Raimondo G., Levrero M. (2009). Nuclear HBx binds the HBV minichromosome and modifies the epigenetic regulation of cccDNA function. Proc. Natl. Acad. Sci. USA.

[27] Lucifora J., Arzberger S., Durantel D., Belloni L., Strubin M., Levrero M., Zoulim F., Hantz O., Protzer U. (2011). Hepatitis B virus X protein is essential to initiate and maintain virus replication after infection. J. Hepatol..

[28] Minor M.M., Slagle B.L. (2014). Hepatitis B virus HBx protein interactions with the ubiquitin proteasome system. Viruses.

[29] Slagle B.L., Bouchard M.J. (2016). Hepatitis B Virus X and Regulation of Viral Gene Expression. Cold Spring Harb Perspect. Med..

[30] Tsuge M., Hiraga N., Akiyama R., Tanaka S., Matsushita M., Mitsui F., Abe H., Kitamura S., Hatakeyama T., Kimura T. (2010). HBx protein is indispensable for development of viraemia in human hepatocyte chimeric mice. J. Gen. Virol..

[31] Decorsiere A., Mueller H., van Breugel P.C., Abdul F., Gerossier L., Beran R.K., Livingston C.M., Niu C., Fletcher S.P., Hantz O. (2016). Hepatitis B virus X protein identifies the Smc5/6 complex as a host restriction factor. Nature.

[32] Murphy C.M., Xu Y., Li F., Nio K., Reszka-Blanco N., Li X., Wu Y., Yu Y., Xiong Y., Su L. (2016). Hepatitis B Virus X Protein Promotes Degradation of SMC5/6 to Enhance HBV Replication. Cell. Rep..

[33] Niu C., Livingston C.M., Li L., Beran R.K., Daffis S., Ramakrishnan D., Burdette D., Peiser L., Salas E., Ramos H. (2017). The Smc5/6 Complex Restricts HBV when Localized to ND10 without Inducing an Innate Immune Response and Is Counteracted by the HBV X Protein Shortly after Infection. PLoS ONE.

[34] Livingston C.M., Ramakrishnan D., Strubin M., Fletcher S.P., Beran R.K. (2017). Identifying and Characterizing Interplay between Hepatitis B Virus X Protein and Smc5/6. Viruses.

[35] Saper G., Kler S., Asor R., Oppenheim A., Raviv U., Harries D. (2013). Effect of capsid confinement on the chromatin organization of the SV40 minichromosome. Nucleic Acids Res..

[36] Köck J., Rösler C., Zhang J.J., Blum H.E., Nassal M., Thoma C. (2010). Generation of covalently closed circular DNA of hepatitis B viruses via intracellular recycling is regulated in a virus specific manner. PLoS Pathog..

[37] Birnbaum F., Nassal M. (1990). Hepatitis B virus nucleocapsid assembly: Primary structure requirements in the core protein. J. Virol..

[38] Nassal M. (1992). The arginine-rich domain of the hepatitis B virus core protein is required for pregenome encapsidation and productive viral positive-strand DNA synthesis but not for virus assembly. J. Virol..

[39] Zlotnick A., Venkatakrishnan B., Tan Z., Lewellyn E., Turner W., Francis S. (2015). Core protein: A pleiotropic keystone in the HBV lifecycle. Antiviral Res..

[40] Protzer U. (2015). Hepatitis: Epigenetic control of HBV by HBx protein--releasing the break?. Nat. Rev. Gastroenterol Hepatol..

[41] Rothbart S.B., Strahl B.D. (2014). Interpreting the language of histone and DNA modifications. Biochim. Biophys. Acta.

[42] Zhang T., Cooper S., Brockdorff N. (2015). The interplay of histone modifications—Writers that read. EMBO Rep..

[43] Tropberger P., Mercier A., Robinson M., Zhong W., Ganem D.E., Holdorf M. (2015). Mapping of histone modifications in episomal HBV cccDNA uncovers an unusual chromatin organization amenable to epigenetic manipulation. Proc. Natl. Acad. Sci. USA.

[44] Porterfield J.Z., Dhason M.S., Loeb D.D., Nassal M., Stray S.J., Zlotnick A. (2010). Full-length hepatitis B virus core protein packages viral and heterologous RNA with similarly high levels of cooperativity. J. Virol..

[45] Guo Y., Kang W., Lei X., Li Y., Xiang A., Liu Y., Zhao J., Zhang J., Yan Z. (2012). Hepatitis B viral core protein disrupts human host gene expression by binding to promoter regions. BMC Genom..

[46] Qi Y., Gao Z., Xu G., Peng B., Liu C., Yan H., Yao Q., Sun G., Liu Y., Tang D. (2016). DNA Polymerase kappa is a key cellular factor for the formation of covalently closed circular DNA of Hepatitis B Virus. PLoS Pathog..

[47] Wang G.Z., Wang Y., Goff S.P. (2016). Histones are rapidly loaded onto unintegrated retroviral DNAs soon after nuclear entry. Cell. Host Microbe.

[48] Chen H.S., Kaneko S., Girones R., Anderson R.W., Hornbuckle W.E., Tennant B.C., Cote P.J., Gerin J.L., Purcell R.H., Miller R.H. (1993). The woodchuck hepatitis virus X gene is important for establishment of virus infection in woodchucks. J. Virol..

[49] Königer C., Wingert I., Marsmann M., Rösler C., Beck J., Nassal M. (2014). Involvement of the host DNA-repair enzyme TDP2 in formation of the covalently closed circular DNA persistence reservoir of hepatitis B viruses. Proc. Natl. Acad. Sci. USA.

[50] Köck J., Schlicht H.J. (1993). Analysis of the earliest steps of hepadnavirus replication: Genome repair after infectious entry into hepatocytes does not depend on viral polymerase activity. J. Virol..

[51] Köck J., Baumert T.F., Delaney W.E.T., Blum H.E., von Weizsacker F. (2003). Inhibitory effect of adefovir and lamivudine on the initiation of hepatitis B virus infection in primary tupaia hepatocytes. Hepatology.

[52] Delmas J., Schorr O., Jamard C., Gibbs C., Trepo C., Hantz O., Zoulim F. (2002). Inhibitory effect of adefovir on viral DNA synthesis and covalently closed circular DNA formation in duck hepatitis B virus-infected hepatocytes in vivo and in vitro. Antimicrob. Agents Chemother..

[53] Moraleda G., Saputelli J., Aldrich C.E., Averett D., Condreay L., Mason W.S. (1997). Lack of effect of antiviral therapy in nondividing hepatocyte cultures on the closed circular DNA of woodchuck hepatitis virus. J. Virol..

[54] Hantz O., Parent R., Durantel D., Gripon P., Guguen-Guillouzo C., Zoulim F. (2009). Persistence of the hepatitis B virus covalently closed circular DNA in HepaRG human hepatocyte-like cells. J. Gen. Virol..

[55] Xia Y., Carpentier A., Cheng X., Block P.D., Zhao Y., Zhang Z., Protzer U., Liang T.J. (2017). Human stem cell-derived hepatocytes as a model for hepatitis B virus infection, spreading and virus-host interactions. J. Hepatol..

[56] Pommier Y., Sun Y., Huang S.N., Nitiss J.L. (2016). Roles of eukaryotic topoisomerases in transcription, replication and genomic stability. Nat. Rev. Mol. Cell. Biol..

[57] Zhang Y.Y., Zhang B.H., Theele D., Litwin S., Toll E., Summers J. (2003). Single-cell analysis of covalently closed circular DNA copy numbers in a hepadnavirus-infected liver. Proc. Natl. Acad. Sci. USA.

[58] Zhu Y., Yamamoto T., Cullen J., Saputelli J., Aldrich C.E., Miller D.S., Litwin S., Furman P.A., Jilbert A.R., Mason W.S. (2001). Kinetics of hepadnavirus loss from the liver during inhibition of viral DNA synthesis. J. Virol..

[59] Summers J., Smith P.M., Horwich A.L. (1990). Hepadnavirus envelope proteins regulate covalently closed circular DNA amplification. J. Virol..

[60] Summers J., Smith P.M., Huang M.J., Yu M.S. (1991). Morphogenetic and regulatory effects of mutations in the envelope proteins of an avian hepadnavirus. J. Virol..

[61] Tuttleman J.S., Pourcel C., Summers J. (1986). Formation of the pool of covalently closed circular viral DNA in hepadnavirus-infected cells. Cell.

[62] Werle-Lapostolle B., Bowden S., Locarnini S., Wursthorn K., Petersen J., Lau G., Trepo C., Marcellin P., Goodman Z., Delaney W.E.T. (2004). Persistence of cccDNA during the natural history of chronic hepatitis B and decline during adefovir dipivoxil therapy. Gastroenterology.

[63] Volz T., Allweiss L., Ben M.M., Warlich M., Lohse A.W., Pollok J.M., Alexandrov A., Urban S., Petersen J., Lutgehetmann M. (2013). The entry inhibitor Myrcludex-B efficiently blocks intrahepatic virus spreading in humanized mice previously infected with hepatitis B virus. J. Hepatol..

[64] Sun D., Nassal M. (2006). Stable HepG2- and Huh7-based human hepatoma cell lines for efficient regulated expression of infectious hepatitis B virus. J. Hepatol..

[65] Lentz T.B., Loeb D.D. (2011). Roles of the envelope proteins in the amplification of covalently closed circular DNA and completion of synthesis of the plus-strand DNA in hepatitis B virus. J. Virol..

[66] Iannacone M., Guidotti L.G. (2015). Mouse models of hepatitis B virus pathogenesis. Cold Spring Harb. Perspect. Med..

[67] Cai D., Nie H., Yan R., Guo J.T., Block T.M., Guo H. (2013). A southern blot assay for detection of hepatitis B virus covalently closed circular DNA from cell cultures. Methods Mol. Biol..

[68] Chisari F.V., Mason W.S., Seeger C. (2014). Virology. Comment on “Specific and nonhepatotoxic degradation of nuclear hepatitis B virus cccDNA”. Science.

[69] Xia Y., Lucifora J., Reisinger F., Heikenwalder M., Protzer U. (2014). Virology. Response to Comment on “Specific and nonhepatotoxic degradation of nuclear hepatitis B virus cccDNA”. Science.

[70] Allweiss L., Volz T., Lutgehetmann M., Giersch K., Bornscheuer T., Lohse A.W., Petersen J., Ma H., Klumpp K., Fletcher S.P. (2014). Immune cell responses are not required to induce substantial hepatitis B virus antigen decline during pegylated interferon-alpha administration. J. Hepatol..

[71] Zhang X., Lu W., Zheng Y., Wang W., Bai L., Chen L., Feng Y., Zhang Z., Yuan Z. (2016). In situ analysis of intrahepatic virological events in chronic hepatitis B virus infection. J. Clin. Investig..

[72] Xia Y., Stadler D., Ko C., Protzer U. (2017). Analyses of HBV cccDNA Quantification and Modification. Methods Mol. Biol..

[73] Sayers J.R., Eckstein F. (1991). A single-strand specific endonuclease activity copurifies with overexpressed T5 D15 exonuclease. Nucleic Acids Res..

[74] Mu D., Yan L., Tang H., Liao Y. (2015). A sensitive and accurate quantification method for the detection of hepatitis B virus covalently closed circular DNA by the application of a droplet digital polymerase chain reaction amplification system. Biotechnol. Lett..

[75] Suspene R., Thiers V., Vartanian J.P., Wain-Hobson S. (2016). PCR mediated recombination impacts the analysis of hepatitis B Virus covalently closed circular DNA. Retrovirology.

[76] Cui X., McAllister R., Boregowda R., Sohn J.A., Cortes Ledesma F., Caldecott K.W., Seeger C., Hu J. (2015). Does tyrosyl DNA phosphodiesterase-2 play a role in hepatitis B virus genome repair?. PLoS ONE.

[77] Ladner S.K., Otto M.J., Barker C.S., Zaifert K., Wang G.H., Guo J.T., Seeger C., King R.W. (1997). Inducible expression of human hepatitis B virus (HBV) in stably transfected hepatoblastoma cells: A novel system for screening potential inhibitors of HBV replication. Antimicrob Agents Chemother.

[78] Cai D., Mills C., Yu W., Yan R., Aldrich C.E., Saputelli J.R., Mason W.S., Xu X., Guo J.T., Block T.M. (2012). Identification of disubstituted sulfonamide compounds as specific inhibitors of hepatitis B virus covalently closed circular DNA formation. Antimicrob. Agents Chemother..

[79] Döring T., Prange R. (2015). Rab33B and its autophagic Atg5/12/16L1 effector assist in hepatitis B virus naked capsid formation and release. Cell. Microbiol..

[80] Cai D., Wang X., Yan R., Mao R., Liu Y., Ji C., Cuconati A., Guo H. (2016). Establishment of an inducible HBV stable cell line that expresses cccDNA-dependent epitope-tagged HBeAg for screening of cccDNA modulators. Antiviral Res..

[81] Guo X., Chen P., Hou X., Xu W., Wang D., Wang T.Y., Zhang L., Zheng G., Gao Z.L., He C.Y. (2016). The recombined cccDNA produced using minicircle technology mimicked HBV genome in structure and function closely. Sci. Rep..

[82] Li F., Cheng L., Murphy C.M., Reszka-Blanco N.J., Wu Y., Chi L., Hu J., Su L. (2016). Minicircle HBV cccDNA with a Gaussia luciferase reporter for investigating HBV cccDNA biology and developing cccDNA-targeting drugs. Sci. Rep..

[83] Gripon P., Diot C., Theze N., Fourel I., Loreal O., Brechot C., Guguen-Guillouzo C. (1988). Hepatitis B virus infection of adult human hepatocytes cultured in the presence of dimethyl sulfoxide. J. Virol..

[84] Köck J., Nassal M., MacNelly S., Baumert T.F., Blum H.E., von Weizsäcker F. (2001). Efficient infection of primary tupaia hepatocytes with purified human and woolly monkey hepatitis B virus. J. Virol..

[85] Von Weizsäcker F., Köck J., MacNelly S., Ren S., Blum H.E., Nassal M. (2004). The tupaia model for the study of hepatitis B virus: Direct infection and HBV genome transduction of primary tupaia hepatocytes. Methods Mol. Med..

[86] Allweiss L., Dandri M. (2016). Experimental in vitro and in vivo models for the study of human hepatitis B virus infection. J. Hepatol..

[87] Gripon P., Rumin S., Urban S., Le Seyec J., Glaise D., Cannie I., Guyomard C., Lucas J., Trepo C., Guguen-Guillouzo C. (2002). Infection of a human hepatoma cell line by hepatitis B virus. Proc. Natl. Acad. Sci. USA.

[88] Zhang Z., Torii N., Hu Z., Jacob J., Liang T.J. (2001). X-deficient woodchuck hepatitis virus mutants behave like attenuated viruses and induce protective immunity in vivo. J. Clin. Investig..

[89] Zoulim F., Saputelli J., Seeger C. (1994). Woodchuck hepatitis virus X protein is required for viral infection in vivo. J. Virol..

[90] Marion M.J., Hantz O., Durantel D. (2010). The HepaRG cell line: Biological properties and relevance as a tool for cell biology, drug metabolism, and virology studies. Methods Mol. Biol..

[91] Li W., Urban S. (2016). Entry of hepatitis B and hepatitis D virus into hepatocytes: Basic insights and clinical implications. J. Hepatol..

[92] Verrier E.R., Colpitts C.C., Schuster C., Zeisel M.B., Baumert T.F. (2016). Cell Culture Models for the Investigation of Hepatitis B and D Virus Infection. Viruses.

[93] Bartenschlager R., Pietschmann T. (2005). Efficient hepatitis C virus cell culture system: What a difference the host cell makes. Proc. Natl. Acad. Sci. USA.

[94] Shlomai A., Schwartz R.E., Ramanan V., Bhatta A., de Jong Y.P., Bhatia S.N., Rice C.M. (2014). Modeling host interactions with hepatitis B virus using primary and induced pluripotent stem cell-derived hepatocellular systems. Proc. Natl. Acad. Sci. USA.

[95] Li Y., Li L.F., Yu S., Wang X., Zhang L., Yu J., Xie L., Li W., Ali R., Qiu H.J. (2016). Applications of Replicating-Competent Reporter-Expressing Viruses in Diagnostic and Molecular Virology. Viruses.

[96] Protzer U., Nassal M., Chiang P.W., Kirschfink M., Schaller H. (1999). Interferon gene transfer by a hepatitis B virus vector efficiently suppresses wild-type virus infection. Proc. Natl. Acad. Sci. USA.

[97] Li B., Sun S., Li M., Cheng X., Li H., Kang F., Kang J., Dornbrack K., Nassal M., Sun D. (2016). Suppression of hepatitis B virus antigen production and replication by wild-type HBV dependently replicating HBV shRNA vectors in vitro and in vivo. Antiviral Res..

[98] Wang Z., Wu L., Cheng X., Liu S., Li B., Li H., Kang F., Wang J., Xia H., Ping C. (2013). Replication-competent infectious hepatitis B virus vectors carrying substantially sized transgenes by redesigned viral polymerase translation. PLoS ONE.

[99] Ryan E.L., Hollingworth R., Grand R.J. (2016). Activation of the DNA damage response by RNA viruses. Biomolecules.

[100] Weitzman M.D., Lilley C.E., Chaurushiya M.S. (2010). Genomes in conflict: Maintaining genome integrity during virus infection. Annu. Rev. Microbiol..

[101] Weitzman M.D., Weitzman J.B. (2014). What’s the damage? The impact of pathogens on pathways that maintain host genome integrity. Cell Host Microbe.

[102] Luftig M.A. (2014). Viruses and the DNA damage response: Activation and antagonism. Annu. Rev. Virol..

[103] Dempsey A., Bowie A.G. (2015). Innate immune recognition of DNA: A recent history. Virology.

[104] Lindahl T., Barnes D.E. (2000). Repair of endogenous DNA damage. Cold Spring Harb. Symp. Quant. Biol..

[105] Pateras I.S., Havaki S., Nikitopoulou X., Vougas K., Townsend P.A., Panayiotidis M.I., Georgakilas A.G., Gorgoulis V.G. (2015). The DNA damage response and immune signaling alliance: Is it good or bad? Nature decides when and where. Pharmacol. Ther..

[106] Schreiner S., Wimmer P., Dobner T. (2012). Adenovirus degradation of cellular proteins. Future Microbiol..

[107] Ceccaldi R., Rondinelli B., D’Andrea A.D. (2016). Repair pathway choices and consequences at the double-strand break. Trends Cell. Biol..

[108] Davis A.J., Chen B.P., Chen D.J. (2014). DNA-PK: A dynamic enzyme in a versatile DSB repair pathway. DNA Repair (Amst).

[109] Hustedt N., Durocher D. (2016). The control of DNA repair by the cell cycle. Nat. Cell. Biol..

[110] Hollingworth R., Grand R.J. (2015). Modulation of DNA damage and repair pathways by human tumour viruses. Viruses.

[111] Paull T.T. (2015). Mechanisms of ATM Activation. Annu. Rev. Biochem..

[112] Lavin M.F., Kozlov S., Gatei M., Kijas A.W. (2015). ATM-Dependent Phosphorylation of All Three Members of the MRN Complex: From Sensor to Adaptor. Biomolecules.

[113] Bakkenist C.J., Kastan M.B. (2015). Chromatin perturbations during the DNA damage response in higher eukaryotes. DNA Repair (Amst).

[114] Cimprich K.A., Cortez D. (2008). ATR: An essential regulator of genome integrity. Nat. Rev. Mol. Cell. Biol..

[115] Nam E.A., Cortez D. (2011). ATR signalling: More than meeting at the fork. Biochem. J..

[116] Matt S., Hofmann T.G. (2016). The DNA damage-induced cell death response: A roadmap to kill cancer cells. Cell. Mol. Life Sci..

[117] Nakad R., Schumacher B. (2016). DNA damage response and immune defense: Links and mechanisms. Front. Genet..

[118] Deng X., Xu P., Zou W., Shen W., Peng J., Liu K., Engelhardt J.F., Yan Z., Qiu J. (2017). DNA damage signaling is required for replication of human bocavirus 1 DNA in dividing HEK293 cells. J. Virol..

[119] Shah G.A., O’Shea C.C. (2015). Viral and cellular genomes activate distinct DNA damage responses. Cell.

[120] Burgess R.C., Misteli T. (2015). Not all DDRs are created equal: Non-canonical DNA damage responses. Cell.

[121] Luo Y., Qiu J. (2013). Parvovirus infection-induced DNA damage response. Future Virol..

[122] McFadden K., Luftig M.A. (2013). Interplay between DNA tumor viruses and the host DNA damage response. Curr. Top. Microbiol. Immunol..

[123] Xiaofei E., Kowalik T.F. (2014). The DNA damage response induced by infection with human cytomegalovirus and other viruses. Viruses.

[124] Bregnard C., Benkirane M., Laguette N. (2014). DNA damage repair machinery and HIV escape from innate immune sensing. Front. Microbiol..

[125] McKinney C.C., Hussmann K.L., McBride A.A. (2015). The Role of the DNA Damage Response throughout the Papillomavirus Life Cycle. Viruses.

[126] Anacker D.C., Moody C.A. (2016). Modulation of the DNA damage response during the life cycle of human papillomaviruses. Virus Res..

[127] Ma Z., Damania B. (2016). The cGAS-STING Defense Pathway and Its Counteraction by Viruses. Cell Host Microbe.

[128] Sung W.K., Zheng H., Li S., Chen R., Liu X., Li Y., Lee N.P., Lee W.H., Ariyaratne P.N., Tennakoon C. (2012). Genome-wide survey of recurrent HBV integration in hepatocellular carcinoma. Nat. Genet..

[129] Mason W.S., Gill U.S., Litwin S., Zhou Y., Peri S., Pop O., Hong M.L., Naik S., Quaglia A., Bertoletti A. (2016). HBV DNA Integration and Clonal Hepatocyte Expansion in Chronic Hepatitis B Patients Considered Immune Tolerant. Gastroenterology.

[130] Kennedy P.T.F., Litwin S., Dolman G.E., Bertoletti A., Mason W.S. (2017). Immune Tolerant Chronic Hepatitis B: The Unrecognized Risks. Viruses.

[131] Bill C.A., Summers J. (2004). Genomic DNA double-strand breaks are targets for hepadnaviral DNA integration. Proc. Natl. Acad. Sci. USA.

[132] Zhao L.H., Liu X., Yan H.X., Li W.Y., Zeng X., Yang Y., Zhao J., Liu S.P., Zhuang X.H., Lin C. (2016). Genomic and oncogenic preference of HBV integration in hepatocellular carcinoma. Nat. Commun..

[133] Yang W., Summers J. (1995). Illegitimate replication of linear hepadnavirus DNA through nonhomologous recombination. J. Virol..

[134] Yang W., Summers J. (1998). Infection of ducklings with virus particles containing linear double-stranded duck hepatitis B virus DNA: Illegitimate replication and reversion. J. Virol..

[135] Sloan R.D., Wainberg M.A. (2011). The role of unintegrated DNA in HIV infection. Retrovirology.

[136] Guo H., Xu C., Zhou T., Block T.M., Guo J.T. (2012). Characterization of the host factors required for hepadnavirus covalently closed circular (ccc) DNA formation. PLoS ONE.

[137] Deriano L., Roth D.B. (2013). Modernizing the nonhomologous end-joining repertoire: Alternative and classical NHEJ share the stage. Annu. Rev. Genet..

[138] Stracker T.H., Carson C.T., Weitzman M.D. (2002). Adenovirus oncoproteins inactivate the Mre11-Rad50-NBS1 DNA repair complex. Nature.

[139] Chung Y.L., Tsai T.Y. (2009). Promyelocytic leukemia nuclear bodies link the DNA damage repair pathway with hepatitis B virus replication: Implications for hepatitis B virus exacerbation during chemotherapy and radiotherapy. Mol. Cancer Res..

[140] Ko H.L., Ren E.C. (2011). Novel poly (ADP-ribose) polymerase 1 binding motif in hepatitis B virus core promoter impairs DNA damage repair. Hepatology.

[141] Chung Y.L. (2013). Defective DNA damage response and repair in liver cells expressing hepatitis B virus surface antigen. FASEB J..

[142] Kitamura K., Wang Z., Chowdhury S., Simadu M., Koura M., Muramatsu M. (2013). Uracil DNA glycosylase counteracts APOBEC3G-induced hypermutation of hepatitis B viral genomes: Excision repair of covalently closed circular DNA. PLoS Pathog..

[143] Higgs M.R., Chouteau P., Lerat H. (2014). ‘Liver let die’: Oxidative DNA damage and hepatotropic viruses. J. Gen. Virol..

[144] Ricardo-Lax I., Ramanan V., Michailidis E., Shamia T., Reuven N., Rice C.M., Shlomai A., Shaul Y. (2015). Hepatitis B virus induces RNR-R2 expression via DNA damage response activation. J. Hepatol..

[145] Hollingworth R., Skalka G.L., Stewart G.S., Hislop A.D., Blackbourn D.J., Grand R.J. (2015). Activation of DNA damage response pathways during lytic replication of KSHV. Viruses.

[146] Slagle B.L., Andrisani O.M., Bouchard M.J., Lee C.G., Ou J.H., Siddiqui A. (2015). Technical standards for hepatitis B virus X protein (HBx) research. Hepatology.

[147] Levrero M., Zucman-Rossi J. (2016). Mechanisms of HBV-induced hepatocellular carcinoma. J. Hepatol..

[148] Zhang T., Xie N., He W., Liu R., Lei Y., Chen Y., Tang H., Liu B., Huang C., Wei Y. (2013). An integrated proteomics and bioinformatics analyses of hepatitis B virus X interacting proteins and identification of a novel interactor apoA-I. J. Proteom..

[149] Capovilla A., Arbuthnot P. (2003). Hepatitis B virus X protein does not influence essential steps of nucleotide excision repair effected by human liver extracts. Biochem. Biophys. Res. Commun..

[150] Capovilla A., Carmona S., Arbuthnot P. (1997). Hepatitis B virus X-protein binds damaged DNA and sensitizes liver cells to ultraviolet irradiation. Biochem. Biophys. Res. Commun..

[151] Jia L., Wang X.W., Harris C.C. (1999). Hepatitis B virus X protein inhibits nucleotide excision repair. Int. J. Cancer.

[152] van de Klundert M.A., van Hemert F.J., Zaaijer H.L., Kootstra N.A. (2012). The hepatitis B virus x protein inhibits thymine DNA glycosylase initiated base excision repair. PLoS ONE.

[153] Truant R., Antunovic J., Greenblatt J., Prives C., Cromlish J.A. (1995). Direct interaction of the hepatitis B virus HBx protein with p53 leads to inhibition by HBx of p53 response element-directed transactivation. J. Virol..

[154] Lee T.H., Elledge S.J., Butel J.S. (1995). Hepatitis B virus X protein interacts with a probable cellular DNA repair protein. J. Virol..

[155] Van der Crabben S.N., Hennus M.P., McGregor G.A., Ritter D.I., Nagamani S.C., Wells O.S., Harakalova M., Chinn I.K., Alt A., Vondrova L. (2016). Destabilized SMC5/6 complex leads to chromosome breakage syndrome with severe lung disease. J. Clin. Investig..

[156] Scrima A., Konickova R., Czyzewski B.K., Kawasaki Y., Jeffrey P.D., Groisman R., Nakatani Y., Iwai S., Pavletich N.P., Thoma N.H. (2008). Structural basis of UV DNA-damage recognition by the DDB1-DDB2 complex. Cell.

[157] Stoyanova T., Roy N., Kopanja D., Raychaudhuri P., Bagchi S. (2009). DDB2 (damaged-DNA binding protein 2) in nucleotide excision repair and DNA damage response. Cell. Cycle.

[158] Brown J.S., Jackson S.P. (2015). Ubiquitylation, neddylation and the DNA damage response. Open Biol..

[159] Hrecka K., Hao C., Shun M.C., Kaur S., Swanson S.K., Florens L., Washburn M.P., Skowronski J. (2016). HIV-1 and HIV-2 exhibit divergent interactions with HLTF and UNG2 DNA repair proteins. Proc. Natl. Acad. Sci. USA.

[160] Wu Y., Zhou X., Barnes C.O., DeLucia M., Cohen A.E., Gronenborn A.M., Ahn J., Calero G. (2016). The DDB1-DCAF1-Vpr-UNG2 crystal structure reveals how HIV-1 Vpr steers human UNG2 toward destruction. Nat. Struct. Mol. Biol..

[161] Ballana E., Este J.A. (2015). SAMHD1: At the crossroads of cell proliferation, immune responses, and virus restriction. Trends Microbiol..

[162] Li T., Robert E.I., van Breugel P.C., Strubin M., Zheng N. (2010). A promiscuous alpha-helical motif anchors viral hijackers and substrate receptors to the CUL4-DDB1 ubiquitin ligase machinery. Nat. Struct. Mol. Biol..

[163] Guerrieri F., Belloni L., D’Andrea D., Pediconi N., Le Pera L., Testoni B., Scisciani C., Floriot O., Zoulim F., Tramontano A. (2017). Genome-wide identification of direct HBx genomic targets. BMC Genom..

[164] Pommier Y., Huang S.Y., Gao R., Das B.B., Murai J., Marchand C. (2014). Tyrosyl-DNA-phosphodiesterases (TDP1 and TDP2). DNA Repair (Amst).

[165] Menon V., Povirk L.F. (2016). End-processing nucleases and phosphodiesterases: An elite supporting cast for the non-homologous end joining pathway of DNA double-strand break repair. DNA Repair (Amst).

[166] Pillaire M.J., Betous R., Hoffmann J.S. (2014). Role of DNA polymerase kappa in the maintenance of genomic stability. Mol. Cell. Oncol..

[167] Zhao L., Washington M.T. (2017). Translesion Synthesis: Insights into the Selection and Switching of DNA Polymerases. Genes.

[168] Kinoshita W., Ogura N., Watashi K., Wakita T. (2017). Host factor PRPF31 is involved in cccDNA production in HBV-replicating cells. Biochem. Biophys. Res. Commun..

[169] Baumert T.F., Verrier E.R., Nassal M., Chung R.T., Zeisel M.B. (2015). Host-targeting agents for treatment of hepatitis B virus infection. Curr. Opin. Virol..

[170] Guo J.T., Guo H. (2015). Metabolism and function of hepatitis B virus cccDNA: Implications for the development of cccDNA-targeting antiviral therapeutics. Antiviral Res..

[171] Testoni B., Durantel D., Zoulim F. (2017). Novel targets for hepatitis B virus therapy. Liver Int..

[172] Faure-Dupuy S., Lucifora J., Durantel D. (2017). Interplay between the hepatitis B virus and innate immunity: From an understanding to the development of therapeutic concepts. Viruses.

[173] Liu Y., Li J., Chen J., Li Y., Wang W., Du X., Song W., Zhang W., Lin L., Yuan Z. (2015). Hepatitis B virus polymerase disrupts K63-linked ubiquitination of STING to block innate cytosolic DNA-sensing pathways. J. Virol..

[174] Luangsay S., Gruffaz M., Isorce N., Testoni B., Michelet M., Faure-Dupuy S., Maadadi S., Ait-Goughoulte M., Parent R., Rivoire M. (2015). Early inhibition of hepatocyte innate responses by hepatitis B virus. J. Hepatol..

